# Intranasal Rapamycin Rescues Mice from Staphylococcal Enterotoxin B-Induced Shock 

**DOI:** 10.3390/toxins4090718

**Published:** 2012-09-18

**Authors:** Teresa Krakauer, Marilyn Buckley

**Affiliations:** Integrated Toxicology Division, U.S. Army Medical Research Institute of Infectious Diseases, Fort Detrick, MD 21702, USA; Email: marilyn.buckley@us.army.mil

**Keywords:** intranasal rapamycin, staphylococcal enterotoxin B, shock

## Abstract

Staphylococcal enterotoxin B (SEB) and related exotoxins produced by *Staphylococcus aureus *are potent activators of the immune system and cause toxic shock in humans. Currently there is no effective treatment except for the use of intravenous immunoglobulins administered shortly after SEB exposure. Intranasal SEB induces long-lasting lung injury which requires prolonged drug treatment. We investigated the effects of rapamycin, an immunosuppressive drug used to prevent graft rejection, by intranasal administration in a lethal mouse model of SEB-induced shock. The results show that intranasal rapamycin alone delivered as late as 17 h after SEB protected 100% of mice from lethal shock. Additionally, rapamycin diminished the weight loss and temperature fluctuations elicited by SEB. Intranasal rapamycin attenuated lung MCP-1, IL-2, IL-6, and IFNγ by 70%, 30%, 64%, and 68% respectively. Furthermore, short courses (three doses) of rapamycin were sufficient to block SEB-induced shock. Intranasal rapamycin represents a novel use of an immunosuppressant targeting directly to site of toxin exposure, reducing dosages needed and allowing a wider therapeutic window.

## 1. Introduction

Staphylococcal enterotoxin B (SEB) and structurally related exotoxins are bacterial virulence factors that cause a variety of diseases in humans, ranging from food poisoning, autoimmune diseases, and toxic shock [1,2,3,4,5,6,7]. These toxins bind directly to the major histocompatibility complex (MHC) class II molecules on antigen-presenting cells and specific Vβ regions of the T-cell receptors [8,9,10,11,12]. Staphylococcal exotoxins (SE) are called superantigens due to their ability to polyclonally activate T cells at picomolar concentrations [7,10,13]. Their interactions with cells of the immune system result in a massive release of proinflammatory cytokines and chemokines [5,7,14]. These proinflammatory mediators enhance leukocyte migration, promote tissue injury, and coagulation [15]. In particular, tumor necrosis factor α (TNFα), interleukin 1 (IL-1) and IFNγ, are pathogenic at high concentrations *in vivo* and are responsible for fever and toxic shock induced by SE [16,17,18,19,20].

In humans, toxic shock syndrome is characterized by fever, hypotension, desquamation of skin, and dysfunction of multiple organ systems [1,4,6]. Humans are very sensitive to SEB intoxication and low doses cause lethal shock, especially via the respiratory route [21]. There is currently no effective therapeutic for treating SEB-induced shock except for the use of intravenous immunoglobulins which must be administered close to the time of toxin exposure [22]. Various murine models were used to develop therapeutics to mitigate SEB-induced shock, although mice are poor responders to SEB due to low affinity of these toxins to mouse MHC class II [9,11]. The most common murine models used rely on the use of sensitizing agents such as D-galactosamine, actinomycin D, lipopolysaccharide (LPS), or viruses to amplify the responses to SEB in toxic shock models [23,24,25]. Transgenic mice expressing human MHC class II were found to be a better animal model for examining the biological effects of superantigens, as they respond to toxins due to the higher affinity binding of SEs to human MHC class II molecules [26,27]. An alternative murine model of toxic shock using two low doses of SEB without the use of confounding sensitizing agents was developed recently [28]. In this “SEB-only” toxic shock model, SEB was administered intranasally and another dose of SEB was strategically given 2 h later by intraperitoneal (i.p.) or intranasal (i.n.) routes to induce systemic and pulmonary inflammation with lethality as an endpoint. 

We described in this study the effect of intranasal rapamycin, a FDA-approved immunosuppressant for kidney transplantation [29], in rescuing mice from SEB-induced shock. Rapamycin binds intracellularly to FK506-binding proteins, specifically FKBP12, the rapamycin-FKBP12 complex then binds to a distinct molecular target called mammalian target of rapamycin (mTOR) and this signaling pathway regulates metabolism as well as immune function [30]. Rapamycin suppresses T cell proliferation [30] and also upregulates the expansion of regulatory T cells [31]. Thus, rapamycin has effects on many types of effector T cells and is likely to be useful in mitigating SEB-activated immune responses. 

## 2. Results and Discussion

### 2.1. Therapeutic Window of Rapamycin Treatment

**Figure 1:**
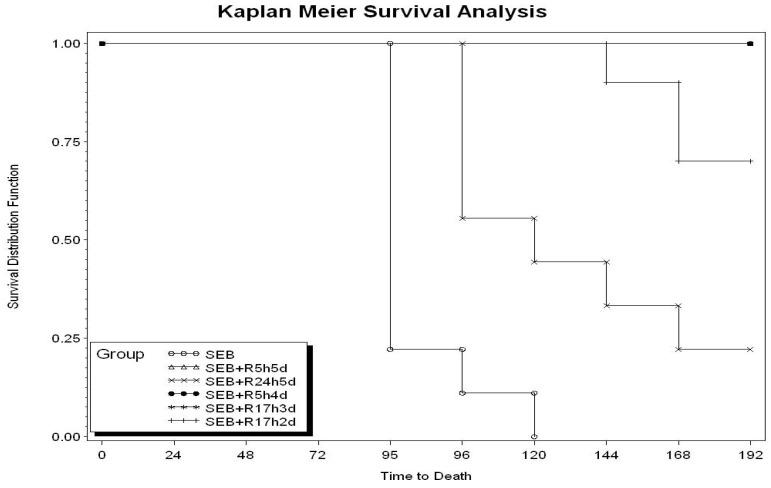
Survival analysis of Staphylococcal enterotoxin B (SEB)-exposed mice treated with intranasal rapamycin. Number of animals and schedule of treatment are identical to those presented in Table 1.

We previously established that rapamycin was effective in attenuating the biological effects of SEB *in vitro* and that multiple dosing schedule of intraperitoneal rapamycin protected mice from SEB-induced shock [32]. Due to the potency of rapamycin by the i.p. route, we investigated if lower doses of rapamycin administered only by the intranasal route would be protective against SEB-induced toxic shock. We explored the therapeutic window of treatment by administrating rapamycin at increasing intervals after SEB exposure. Intranasal administration of rapamycin (0.16 mg/kg) at 5 h after SEB followed by the same dose i.n. at 24, 48, 72, 96 h (R5h5d) protected mice 100% (Table 1). Only 22% survival was recorded if intranasal rapamycin was delayed to 24 h after SEB (R245d). However, starting rapamycin at 5 h after SEB exposure but using one less dose was 100% effective (R5h4d). Importantly, low intranasal doses of rapamycin administered as late as 17 h after SEB exposure followed by doses at 23, 41 h was still 100% protective (R17h3d). The last dose at 41 h was necessary using this schedule of treatment, as eliminating this dose yielded only 70% survival. Kaplan Meier survival analysis (Figure 1) shows rapamycin extended survival times even in unprotected animals. Clinical signs of intoxication such as ruffled fur and lethargy observed with SEB-treated mice starting at 72 h were completely absent from the SEB plus rapamycin group. 

**Table 1:** Protective effects of intranasal rapamycin.

Toxin + rapamycin (R) regiment ^a^	Live/Total ^b^
SEB	0/9
SEB + R at 5, 24, 48, 72, 96 h (R5h5d)	9/9
SEB + R at 24, 30, 48, 72, 96 h (R24h5d)	2/9
SEB	0/10
SEB + R at 5, 24, 48, 72 h (R5h4d)	10/10
SEB + R at 17, 23, 41 h (R17h3d)	10/10
SEB + R at 17, 23 h (R17h2d)	7/10

^a^ Rapamycin (0.16 mg/kg) was administered i.n. at the specific time after SEB exposure as indicated. Bovine serum albumin controls (5 μg i.n. plus 2 µg i.p.) yielded no deaths (*n* = 10 animals per group). ^b^ Results obtained with rapamycin groups were statistically significant (except for the SEB + R24h5d group) from SEB groups (*p* < 0.02).

### 2.2. Rapamycin Prevents Hyperthermia in SEB-Induced Shock Model

**Figure 2:**
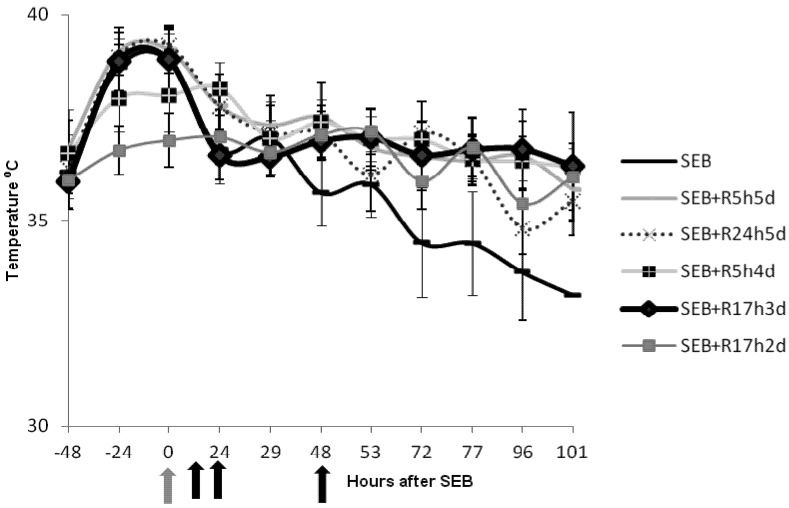
Intranasal rapamycin attenuated the hypothermic response of mice treated with SEB. Body temperatures of mice (*n* = 9 or *n* = 10) exposed to SEB and SEB plus rapamycin (0.16 mg/kg) at different time points after SEB exposure. Group identifiers are identical to those in Table 1. Points represent the means ± SD for each group. Grey arrow indicates time of SEB exposure and black arrows represent time of rapamycin administration for the SEB + R17h3d group.

Additional data were collected regarding temperature fluctuations in mice treated with SEB and those treated with SEB plus intranasal rapamycin given at various times after SEB (Figure 2). Mice given SEB experienced hypothermia starting at 48 h. This hypothermic response, indicating systemic shock that mimicked those found in other murine models [26,33,34], was completely absent in rapamycin-treated SEB-exposed mice. Reducing the duration of treatment with rapamycin to 72 h also protected mice from hyperthermia if treatment was started at 5 h (SEB + R5h4d). However, delaying treatment with rapamycin until 24 h resulted in shock like symptoms and hyperthermia (SEB + R24h5d). We progressively adjusted the time between the exposure of mice to SEB and rapamycin treatment to determine the maximum therapeutic window. A protective regimen of rapamycin starting at 17 h after SEB exposure followed by two other intranasal doses at 23 and 41 h also did not result in hypothermia. Clearly, rapamycin-treated and protected mice had minor temperature changes during the entire observation period.

### 2.3. Rapamycin Prevents Weight Loss in SEB-Induced Shock Model

**Figure 3:**
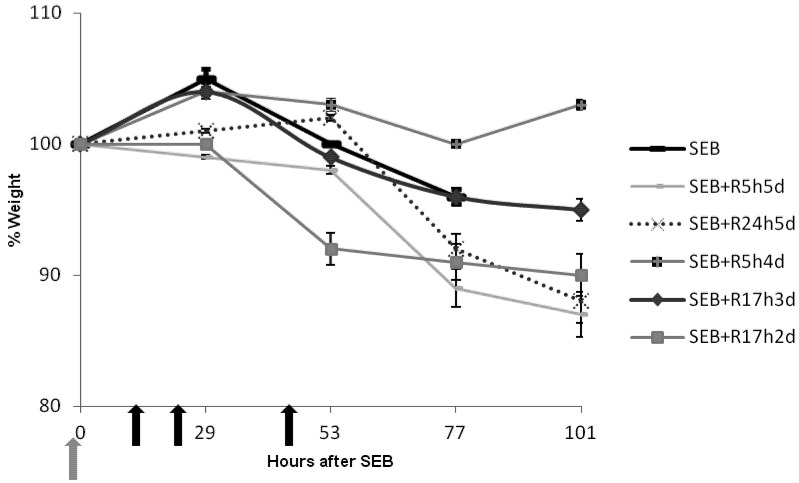
Intranasal rapamycin prevented weight loss in mice treated with SEB. Weights of mice (*n* = 9 or *n* = 10) exposed to SEB and SEB plus rapamycin (0.16 mg/kg) at different time points after SEB exposure. Group identifiers are identical to those in Table 1. Points represent the average % weight change ± SD for each group. Grey arrow indicates time of SEB exposure and black arrows represent time of rapamycin administration for the SEB + R17h3d group.

Weight loss is another prominent indicator of SEB-induced shock in other animal models of superantigen-induced disease [16]. We therefore examined the effect of rapamycin on the weight of animals after intranasal SEB. SEB-exposed mice experienced weight loss of 3%–7% at 53 h with drastic weight reduction over time. Most of the rapamycin-treated, SEB-exposed mice had 1% change in weight with some groups of mice gaining weight at later times (Figure 3). Mice receiving only two doses of rapamycin starting at 17 h (SEB + R17hd2) had 8% weight loss and weight remained at this level after 53 h (70% survival). Protection against temperature and weight fluctuations essentially paralleled the lethality data. 

### 2.4. Rapamycin Attenuates Pulmonary Mediators

**Figure 4:**
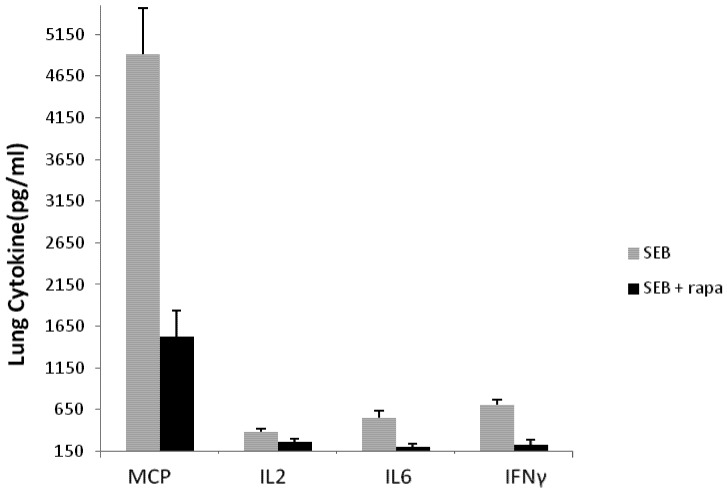
Chemokine and cytokine assessment at 42 h in lungs of mice treated with intranasal rapamycin after SEB challenge. Values represent the mean ± SE for five mice and results are statistically significant (*p* < 0.05) between SEB and SEB + rapamycin group.

Proinflammatory cytokines mediate the lethal and pathological effects of SEB and monocyte chemoattractant protein (MCP-1), interleukin 2 (IL-2), IL-6 and gamma interferon (IFNγ) are critical mediators in various mouse models of SEB-induced shock [19,23,26,28,32]. We next examined the effect of intranasal rapamycin on these pulmonary mediators. Figure 4 shows that high levels of MCP-1, IL-2, IL-6, and IFNγ were present in lung tissue at 42 h post-SEB treatment. In contrast, mice treated with rapamycin at 15 h followed by two additional doses at 21 and 39 h after SEB attenuated MCP-1, IL-2, IL-6, and IFNγ by 70%, 30%, 64%, and 68% respectively. These findings indicate a protective regimen of rapamycin is also effective in reducing the pulmonary mediators critical in initiating cell migration, cell activation, inflammation culminating in lung injury. 

Bacterial superantigens cause toxic shock and contribute to septic complications during infection and only antibodies to superantigens and steroids had proven to be effective when administered early after exposure. The present study demonstrates for the first time that intranasal low dose rapamycin was 100% effective in protecting mice from SEB-mediated shock. Previous studies of drug treatment for SEB- and other superantigen-induced shock models indicate a very narrow therapeutic window of treatment [19,20]. This study indicates a much wider therapeutic window for rapamycin and that a new modality of using low doses of rapamycin even when administered 17 h after SEB was still protective 100% against mortality. Concomitantly, intranasal rapamycin treatment reduced temperature and weight fluctuations in SEB-treated mice.

Excessive release of cytokines contributes to the pathophysiological process of toxic shock as proinflammatory cytokines and chemokines regulate leukocyte migration and promote tissue injury. We previously reported that IL-2 and MCP-1 play a central role in this intranasal model of SEB-induced toxicity and lung inflammation [28]. IFNγ, the prototypic T helper 1 type cytokine produced by activated T cells is a key inflammatory cytokine as it synergizes with other cytokines to promote injury in addition to its anti-viral and anti-proliferative effects. IFNγ was also showed to affect metabolism as anti-IFNγ prevented weight loss in a D-galctosamine-sensitized murine model of SEB-induced shock [35]. Importantly, these critical cytokines were elevated in lung tissue after SEB exposure and were effectively reduced in lungs with rapamycin treatment.

Based on the data presented, we propose that the mTOR signaling pathway plays an important role in the pathophysiology of SEB-mediated shock. The serine-threonine kinase, mTOR senses nutrient/growth factor inputs and cellular ATP levels via growth factor receptors and AMP-activated protein kinase, respectively [30]. In a separate pathway, IL-2 and IFNγ receptor signaling activates phosphoinositide 3-kinase (PI3K) which acts upstream of mTOR and provides a critical link between cytokine receptor signaling and energy homeostasis. mTOR complex 1 (mTORC1), one of two key proteins of mTOR signaling is sensitive to rapamycin. Rapamycin acts by binding to FKBP12, an immunophilin, blocking mTOC1 activity and inhibiting cell cycle progression and protein synthesis. In this regard, it is interesting that FKBP12 is also found in abundance in neuronal cells and blocking FKBP12 was reported to reverse neuronal degeneration [36]. Bidirectional crosstalk between the neuroendocrine and the immune system is critical in the elimination of pathogens and proinflammatory cytokines are known to act on the hypothalamus to modulate neuronal pathways [37]. Future experiments should address if neuroimmunophilins are involved in the SEB-intoxication process. 

## 3. Experimental Section

### 3.1. Materials

Purified SEB was obtained from Toxin Technology (Sarasota, FL, USA). The endotoxin content of these preparations was <1 ng of endotoxin/mg protein, as determined by the Limulus amoebocyte lysate assay (BioWhittaker, Walkersville, MD, USA). Rapamycin and other reagents were from Sigma (St. Louis, MO, USA).

### 3.2. Mouse Model of SEB-Induced Shock

Male C3H/HeJ mice (National Cancer Institute, Frederick, MD, USA), weighing ~20 g each (7–10-weeks old), were housed in conventional microisolator cages. Sterile temperature/identification transponders (IPTT-300, Biomedic Data Systems, Seaford, DE, USA) were implanted subcutaneously into each animal 5–10 days before SEB and temperatures were monitored twice daily, between 8 and 9 a.m. and again between 3 and 4 p.m. A previous study with a murine LPS-potentiated SEB-induced shock model using telemetry to monitor core temperature indicated a good correlation between core and subcutaneous temperature changes [34]. Initial weight of animals was recorded 3–7 days before SEB exposure and weight changes were recorded once daily between 3 and 4 p.m. Temperature and weight were recorded at the same time of the day to avoid diurnal effects. SEB was administered i.n. (50 μL), 5 µg/mouse with a micropipet and i.p. (200 µL), 2 µg/mouse with a tuberculin syringe (26G-3/8-inch needle) between 8 and 9 a.m., immediately after the first temperature measurements of the day. All i.n. doses were administered to mice previously anesthetized with an intramuscular (i.m.)-injected mixture of ketamine (2.4 mg/kg), acepromazine (0.024 mg/kg), and xylazine (0.27 mg/kg). There were 2 h of elapsed time between the first i.n. dose and the second i.p. dose of SEB as this was the optimal time previously determined to cause toxic shock without the use of synergistic agents [28]. Mice exposed to both doses of SEB were hypothermic starting between 48h–72 h and lethal endpoints were recorded up to 192 h after the first toxin dose. Controls consisted of C3H/HeJ mice given two doses of either bovine serum albumin (BSA, Sigma Chemical Corp.) or saline 2 h apart, similar to those of SEB-exposed mice (*i.e.*, by i.n. and i.p.). For therapeutic investigations, mice (*n* = 9 or *n* = 10 mice per group as specified in figures) were given rapamycin i.n. at specific times after intranasal SEB exposure as described for each series of experiment. Intranasal rapamycin (0.16 mg/kg of body weight) in sterile saline was administered with a micropipette at the designated time points. Animals were monitored twice daily for illness and moribund condition for 96 h and as needed. Temperature and weight changes were measured daily up to 96–120 h. Temperature data were calculated as the mean temperature reading ± standard deviation of each group (*n* = 9 or *n* = 10 mice per group). Survival rates were recorded at 192 h. 

Research was conducted under an IACUC approved protocol in compliance with the Animal Welfare Act and other Federal statutes and regulations relating to animals and experiments involving animals and adheres to principles stated in the Guide for the Care and Use of Laboratory Animals, National Research Council, 1996. The facility where this research was conducted is fully accredited by the Association for Assessment and Accreditation of Laboratory Animal Care International.

### 3.3. Cytokine and Chemokine Assays

IL-2, IL-6, and MCP-1 were assayed by ELISA using matched pairs of specific anti-mouse antibodies as described previously [28,32]. Matched pairs of anti-mouse antibodies for cytokines and chemokines, and protein standards were purchased from BD Pharmingen (San Diego, CA, USA) and used in ELISA assays of cytokines according to manufacturer’s protocols. IFNγ was determined using Quantikine ELISA kit from R&D Systems (Minneapolis, MN, USA) as described in manufacturer’s instruction manual.

Whole lungs (*n* = 5 mice per group) were excised from euthanized animals at 42 h post-SEB challenge, weighed, and frozen at −70 °C. Rapamycin treated mice received rapamycin (0.16 mg/kg) intranasally at 15 h after SEB followed by intranasal doses at 21 and 39 h. Immediately before cytokine assays, lungs were thawed and homogenized for 30 min at 4 °C in lysis buffer (Cell Signaling Technology, Danvers, MA, USA) to a 6.9 mg/mL concentration with Complete protease inhibitor (Roche Applied Science, Indianapolis, IN, USA). Homogenates were then centrifuged at 500 × *g* for 10 min, supernatants harvested, and the latter filtered through a 0.45 μM membrane (Millipore, Bedford, MA, USA). Bicinchoninic acid assay (Pierce, Rockford, IL, USA) was used to estimate protein concentrations in lung homogenates and results of lung homogenates were normalized based on protein concentration. 

### 3.4. Statistical Analysis

Data were expressed as the mean ± SE and were analyzed for significant difference by Student’s *t*-test. Statistical comparisons of survival data were performed by Fisher’s exact test with Stata software (Stata Corp., College Station, TX, USA). Differences were considered significant if *P* was <0.05.

## 4. Conclusions

The novel application of intranasal rapamycin represents a new treatment modality for SEB introduced via the respiratory route. This concept of directing drug delivery to site of inflammation and injury has proven to be fast and effective as shown in the use of steroid for treating asthma. In applying this concept, we found low doses of intranasal rapamycin to be effective when administered as late as 17 h after SEB. In comparison, our previous study with the steroid dexamethasone in the same SEB-induced shock model showed that dexamethasone was effective only if administered 5 h after SEB and was not protective when given at 17 h [32]. Furthermore, long courses of dexamethasone up to 96 h were required to prevent shock whereas a much shorter duration of treatment with intranasal rapamycin (41 h) was sufficient to prevent SEB-induced shock. The shorter duration of treatment and delayed start of treatment after SEB exposure with rapamycin is of obvious advantage as currently the only effective treatment with anti-toxin/superantigen or steroid can prevent shock only if treatment is administered early post-SEB exposure. 
